# PEAKQC: periodicity evaluation in single-cell ATAC-seq data for quality assessment

**DOI:** 10.1093/bib/bbaf465

**Published:** 2025-09-16

**Authors:** Jan Detleffsen, Brenton Bruns, Mette Bentsen, Carsten Kuenne, Mario Looso

**Affiliations:** Bioinformatics Core Unit (BCU), Max Planck Institute for Heart and Lung Research, Ludwigstrasse 43, 61231 Bad Nauheim, Hessen, Germany; Translational Hub2 - Cardio-pulmonary systems biology & medicine, Cardio-Pulmonary Institute (CPI), Ludwigstrasse 43, 61231 Bad Nauheim, Hessen, Germany; Bioinformatics Core Unit (BCU), Max Planck Institute for Heart and Lung Research, Ludwigstrasse 43, 61231 Bad Nauheim, Hessen, Germany; Bioinformatics Core Unit (BCU), Max Planck Institute for Heart and Lung Research, Ludwigstrasse 43, 61231 Bad Nauheim, Hessen, Germany; Bioinformatics Core Unit (BCU), Max Planck Institute for Heart and Lung Research, Ludwigstrasse 43, 61231 Bad Nauheim, Hessen, Germany; Bioinformatics Core Unit (BCU), Max Planck Institute for Heart and Lung Research, Ludwigstrasse 43, 61231 Bad Nauheim, Hessen, Germany; Translational Hub2 - Cardio-pulmonary systems biology & medicine, Cardio-Pulmonary Institute (CPI), Ludwigstrasse 43, 61231 Bad Nauheim, Hessen, Germany

**Keywords:** periodicity, scATAC, fragment length distribution, nucleosomal pattern, QC

## Abstract

Chromatin organization guides gene regulatory mechanisms and has been subject of extensive research using chromatin accessibility assays. ATAC-seq is commonly applied to elucidate regulatory regions of the genome at both bulk and single-cell resolutions. However, the analysis of single-cell ATAC-seq data is particularly challenging due to issues such as data sparsity, low signal-to-noise ratios, and the lack of standardized quality control (QC) protocols. While QC based on the fragment length distribution (FLD) represents common practice for bulk analyses, an algorithmic solution that utilizes the full potential of the FLD at the single-cell level is missing. To address this limitation, we introduce the python package PEAKQC, a novel tool that provides a robust metric for identifying high-quality cells. PEAKQC quantifies the deviation of individual cells’ FLD patterns from the expected distribution using a wavelet transformation-based convolution approach. Benchmarking against alternative metrics revealed favorable selection of high-quality cells, facilitating accurate downstream analysis including cell type identification and cluster separation. PEAKQC is readily installable via the Python Package Index and can be seamlessly integrated into existing single-cell analysis frameworks that utilize Python. By providing a robust and scalable solution for single-cell ATAC-seq QC, PEAKQC addresses a significant knowledge gap in the field and proposes FLD patterns as a novel standard for data quality assessment.

## Introduction

Chromatin accessibility assays have been extensively utilized to identify and characterize regulatory regions of the genome that contribute to finely balanced processes of gene activation, gene deactivation, and the permanent closure of DNA regions. These assays include protocols such as DNase-seq [1], FAIRE-seq [2], NOMe-seq [3], MNase-seq [4], or the assay for transposase-accesible chromatin using sequencing (ATAC-seq) [5]. They predominantly rely on fragmentation patterns of DNA, generated by either enzymes or chemical modifications, driven by a stochastic process at accessible DNA sites that are not blocked by nucleosomes or other proteins. Typically, chromatin exists in two primary states, namely euchromatin, characterized by loosely packed nucleosomes and accessible DNA, and heterochromatin, in which tightly packed nucleosomes prevent access to DNA by regulatory proteins [6]. DNase-seq and ATAC-seq were introduced at bulk-level resolution and have recently been adapted toward the single-cell scale [7, 8].

**Figure 1:**
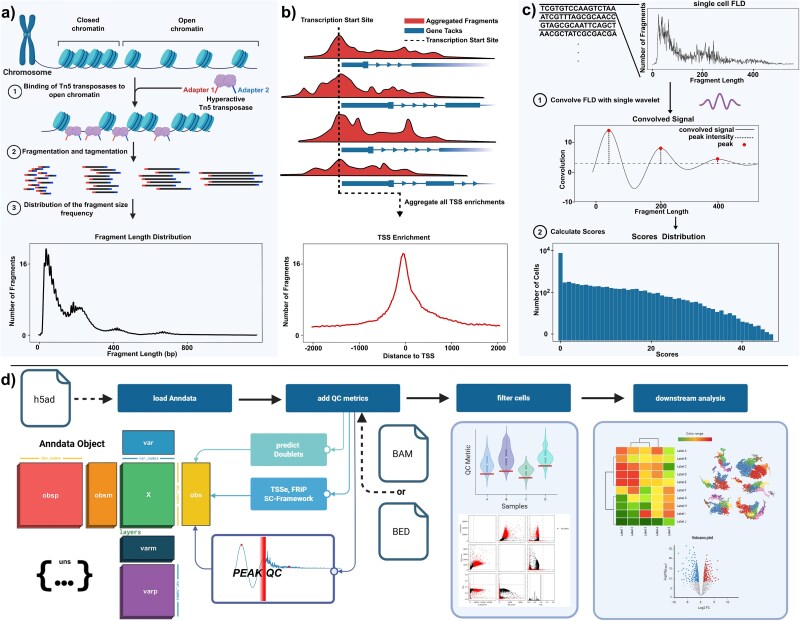
ATAC-seq specific properties. (a) Mechanism creating a distinct pattern observable in the fragment length distribution of an ATAC-seq experiment. This includes binding of Tn5 in euchromatin and resulting fragment lengths from subsequent cleavage. (b) Scheme of TSSe across the whole genome. Tracks show fragments accumulated in relation to exons and introns. (c) Workflow of the PEAKQC algorithm. First FLD of a single cell is calculated from a BAM file or fragment BED file. This is convolved with a custom single wavelet, which enables a more clear representation of the signal. Scores are calculated based on convolution fit. (d) Basic QC workflow integrating PEAKQC starting with an h5ad file. QC metrics such as PEAKQC scores, FRiP, TSSe, and doublet scores are then stored in the observables table for subsequent filtering on these. Downstream analysis is performed on the filtered cells. For most QC metrics, sequence or fragment information is needed and provided by standard formats such as BAM or BED. Schematic of the AnnData object, adapted from Virshup et al. anndata: access and store annotated data matrices*. J Open Sour Softw* 2024;**9**(101):4371. https://doi.org/10.21105/joss.04371 (CC BY 4.0).

ATAC-seq arguably represents the most frequently used protocol to detect chromatin accessibility [9, 10]. It employs Tn5-transposase, resulting in fragments of unequal size which are sequenced and subsequently mapped to a reference genome [5]. When plotting fragments as a function of length frequency, a distinct periodical pattern commonly known as the fragment length distribution (FLD) can be observed (Fig. 1a). This typical pattern results from DNA stretches of fixed length wrapped around histones in a nucleosome. The first peak of about 50 base pairs (bp) originates from fragments of open chromatin and linker DNA. Peaks accounting for larger fragment sizes can be explained by steric hindrance and linker regions which lead to fragment lengths of ~200 bp for mononucleosomes and ~400 bp for dinucleosomes [6]. Theoretically, this pattern can be extended to trinucleosomes and beyond [6, 7].

Reliable identification of regulatory elements such as promoters and enhancers using ATAC-seq critically depends on robust data quality assessment routines. For bulk ATAC-seq, data quality control (QC) standards have been widely established and are divided into QC steps performed on raw reads, such as base quality scoring with tools like FastQC [11], and QC following sequence alignment [10, 12]. Here, we focus on post-alignment QC, which is classified into four main categories:

(i) enrichment of open chromatin signals at certain regions/peaks visible in the signal-to-noise ratio, commonly referred to as transcription start site (TSS) enrichment (Fig. 1b) (TSSe, annotation biased), or the ratio of fragments in peaks (FRiP, unbiased) [13];
(ii) library complexity, determined by total number of unique fragments;
(iii) the ratio of mitochondrial to nuclear DNA;
(iv) FLD (Fig. 1a) [14].
  These measures are broadly accepted standards [10, 13, 14].

To utilize FLD for QC, the nucleosomal pattern is usually inspected visually [10, 12]. Deviations indicate (i) technical errors such as inappropriate Tn5-nuclei ratio, inflated Tn5 incubation time [5] or overcharged cluster density of sequencing lanes [15] and (ii) biological artefacts such as DNA fragmentation due to apoptosis [16] or necrosis [17].

At the single-cell level, QC standards from of bulk ATAC-seq are only partially applicable. Due to data sparsity, the presence of doublet cells, and low signal-to-noise ratios, single-cell data introduces unique challenges [18, 19]. While a number of guidelines exist for single-cell RNA-seq, few guidelines currently address single-cell ATAC-seq (scATAC-seq) QC. Despite recent advances [20], bulk-level guidelines are often directly applied to single cells, as demonstrated by SnapATAC [18], Muon [21], or Preissl *et al*. [22]. Moreover, there is a lack of consensus on effective metrics and limited guidance for selecting reasonable thresholds [14, 23, 24]. However, if details for individual studies are provided, explicit cutoffs considerably differ between datasets, while filtering for outliers is often the only common approach [20]. Reviewing the four prominent scATAC frameworks SnapATAC, Muon, ArchR [25], and Signac [26] reveals substantial variability in available metrics, QC usage, and implementations (Table 1).

**Table 1:** Comparison of QC metrics provided by widely used SC ATAC frameworks

Metric available	Muon	SnapATAC	ArchR	Signac
Library complexity	Total fragment count, total number of features	Uniquely mapped fragments, total number of features	Unique nuclear fragments, not mapping to mito-DNA	Total number of fragments in peaks
Signal-to-noise ratio	TSSe	TSSe, FRiP	TSSe	TSS enrichment, FRIP, ratio reads in genomic blacklist regions
FLD	Mononucleosomal/nucleosome-free ratio (mNSC)	Only plotting on sample level	Only plotting on sample level	Mononucleosomal/nucleosome-free ratio
Mitochondrial DNA ratio	No	Yes	No	No

Given the absence of standardized practices, filtering of scATAC-seq data remains complex and is often driven by personal preferences. Application of FLD for QC on the single-cell level is rare or only roughly estimated when compared to other metrics (Table 1). Due to high numbers of individual FLD patterns (one per each cell), an improved algorithmic solution is required, as visual inspection of a large number of fragment patterns is not feasible. Some tools such as Muon [21] or Signac provide an algorithmic approach by measuring the ratio of nucleosome-spanning fragments [27]. However, this approach only returns a low resolution estimate when compared to a detailed assessment of FLD information by visual inspection. Cusanovich [28] proposed another automated method based on calculating a periodogram to infer actual signals and patterns rather than fragment lengths ratios. However no tool or code was published. Given the fundamental role of FLD in bulk ATAC, we conclude that an algorithmic solution capable of scoring single-cell FLD patterns would significantly enhance scATAC-seq data analysis. Here, we introduce PEAKQC, a Python-based tool that infers cell quality from periodic FLD patterns. PEAKQC utilizes a wavelet based convolution of the FLD to denoise the signal which can subsequently be validated based on the expected periodical pattern.

**Figure 2:**
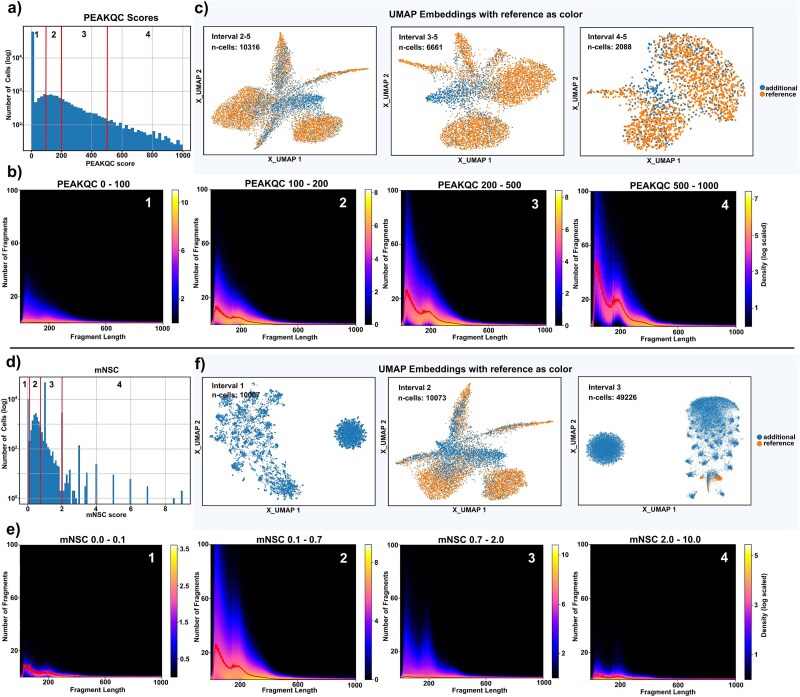
Threshold impacts. (a and d) Distributions of PEAKQC scores and nucleosome signal derived from Muon for the IOBHN sample from CATLAS, categorized into four intervals. (c and f) UMAP embeddings representing subsets of cells grouped by the respective score intervals. (b and e) Density plots of FLDs for single cells.

Of note, all QC metrics described require careful selection of upper and lower cutoffs, to address issues such as doublets, peaks with extreme coverage, or cells with highly fragmented DNA. This motivated the development of a robust QC score that reliable identifies high-quality cells using a single cutoff (see also Result section for muon FLD ratio; Fig. 2). Further, such an optimal score would benefit from a linear scale correlating with signal quality (and not necessarily with e.g. read depth).

We found the PEAKQC-derived cell score robust on a linear scale, mirroring, or partially surpassing other QC metrics. Therefore, we suggest this metric as an integrated alternative for ATAC-seq QC assessment.

## Results

### Package, performance, and workflow

In scATAC-seq analysis, QC can be conducted at multiple stages. PEAKQC is designed for integration into analytical workflows after initial preprocessing steps. It operates on a matrix object containing cell barcodes, alongside a supplementary file bearing fragment size information, thus facilitating barcode-to-fragment associations. PEAKQC utilizes the widely adopted AnnData [29] class object, ensuring compatibility with popular Python-based ecosystems such as Scanpy [30], Episcanpy [24], and Muon [21]. Fragments can be provided in BAM or BED file formats. It is recommended to include only nuclear derived fragments and exclude mitochondrial sequences. Additionally, doublet detection should be performed separately, as this feature is not integrated into PEAKQC.

Despite the widespread use of deep learning for pattern recognition across domains [31, 32], its computational demands and reliance on specialized hardware, such as graphics processing units, pose significant limitations. To facilitate the deployment of PEAKQC on conventional consumer-grade personal computers or laptops, an alternative algorithmic strategy was adopted. The scoring algorithm of PEAKQC is divided into three key stages (Fig. 1c): (i) loading the single-cell FLDs from BAM or BED files, (ii) denoising the FLDs by convolution with a wavelet, and finally (iii) scoring them using a scoring mask. A detailed description is available in Methods section. To evaluate runtime performance, we benchmarked PEAKQC on a virtual machine using two datasets [33] and a setup with 12 CPU cores. Both datasets were subsetted into five runs each to evaluate runtimes. Memory and runtime scaled linearly with input size (see Supplemental Table S1), indicating that PEAKQC can be executed efficiently on standard consumer-grade computing hardware.

In addition to cell scoring, PEAKQC provides a plotting module that enables visualization of individual cell patterns or cell groups via FLD density plots. PEAKQC is available on Github (https://github.com/loosolab/PEAKQC) and on the Python Package Index [34]. It is integrated into the scverse ecosystem (https://scverse.org/packages/#ecosystem) and follows a modular design to facilitate code maintenance. An example workflow integrating PEAKQC is shown in Fig. 1d. Additionally, an example notebook demonstrating PEAKQC usage is available on Github.

### Features, QC benchmarking, and evaluation

This study evaluates and benchmarks PEAKQC’s performance against existing methodologies. Most analyses utilized data from the comprehensive Cis-element ATLAS (CATLAS) database, a large-scale human tissue repository by Zhang *et al*. [19]. The dataset includes raw FASTQ files and preprocessed matrix files with annotated cell types that were filtered by the original authors based on TSSe, the number of unique fragments and doublets. Cells retained after this filtering were defined as the gold standard for benchmarking. To assess generalizability of our approach across diverse cell preparation methods, such as split pooling and microfluidics, we further analyzed two additional datasets generated by 10× Genomics technology.

### Robustness of PEAKQC score and characteristics of FLD-based metrics

Initially, we investigated the characteristics of cells selected by PEAKQC and assessed its ability to robustly identify high-quality cells. We evaluated cells from the right atrium auricular region tissue (Sample ID: IOBHN) subset and subsequently divided them into slices based on increasing PEAKQC score thresholds (Fig. 2a) without imposing an upper cutoff. Thresholds were empirically chosen to yield roughly 4000 cells per cohort. For each slice, a density plot was generated to visualize the average FLD per subset (Fig. 2b, Subsets 1–4). Notably, the observed periodic pattern increases in prominence with rising thresholds, indicating that PEAKQC serves as a reliable and robust quality metric. The linear relationship between the PEAKQC score and the FLD pattern, reflects optimal Tn5 digestion within the underlying cells. PEAKQC employs a multithreaded multinomial downsampling approach to normalize fragment numbers per cell, thereby ensuring independence from fragment numbers both within and across different datasets, provided the sampling number is consistent. Further details on cutoffs are available on Github.

Next, we evaluated how PEAKQC filtering affects downstream scATAC analysis. Utilizing the PEAKQC score thresholds established earlier, we performed three analysis runs, including (i) filtering by score, (ii) removal of mitochondrial and sex chromosome features, (iii) dimensionality reduction using Latent Semantic Indexing [20], and (iv) calculation of a Uniform Manifold Approximation and Projection (UMAP) [35] for two-dimensional visualization. Because PEAKQC correlates strongly with nucleosomal pattern strength, a single cutoff effectively removes all low-quality cells, in contrast to other FLD metrics discussed below.

UMAP plots were annotated to indicate cells overlapping with the CATLAS reference (Fig. 2c). Starting from 73 582 cells, the application of a PEAKQC threshold of 100 reduced the dataset to 10 316 cells. The UMAP visualization revealed distinct clusters and a substantial overlap with the CATLAS reference. However, one central cell cluster was not represented in the CATLAS reference. Increasing the threshold to 200 further reduced the dataset to 6603 cells, still preserving distinct clusters, and slightly improving reference overlap, though a separate non-overlapping cluster remained. At a threshold of 500, clusters were reduced from six to two, suggesting variability in FLD quality across different cell types. Further inspection revealed that certain cell types, such as cardiomyocytes and Fibroblasts, exhibited a pronounced nucleosomal pattern, whereas others displayed a less distinct pattern (Supplemental Fig. S1). Of note, we were not able to link these variances in FLD patterns and scores to specific cell types when comparing different datasets. (Supplemental Fig. S2). As filtering could potentially exclude specific cell types or entire clusters, we checked the impact of moderately filtering with PEAKQC on cells filtered without PEAKQC before. Annotation with the CATLAS reference confirmed that clusters represent biologically meaningful cell types (Supplemental Fig. S3a). Cells with PEAKQC scores below 100 were evenly distributed across clusters (Supplemental Fig. S3b), indicating that no specific population was selectively lost. Additionally, these excluded cells showed weak nucleosomal patterns, supporting their lower quality (Supplemental Fig. S3c). In summary, filtering based on PEAKQC enables a stable reduction in cell numbers, sometimes resulting in the elimination of entire clusters under stringent filtering conditions, while maintaining a consistent overlap with the CATLAS reference. Moreover, a subset of cells that passed the PEAKQC filtering criteria but were not captured by the gold standard reference was identified, warranting further investigation (see below).

To evaluate the robustness and filtering properties of an alternative FLD score, a comparative analysis was conducted utilizing the nucleosome signal of Muon (mNSC). By subdividing cells into discrete intervals (Fig. 2d), we generated a series of FLD density plots (Fig. 2e) that significantly differed from those obtained via PEAKQC score. Notably the first interval (mNSC score between 0 and 0.1) failed to display a discernible periodic pattern. The second interval yielded a periodic, albeit diffuse pattern that bore resemblance to a composite of intervals 2 and 3 of the PEAKQC-based subsets. Interestingly, for the third and fourth intervals, the pattern became less prominent, accompanied by a decrease in fragment counts. These findings suggest that the mNSC score exhibits a non-linear scoring behavior. A cellwise comparison of the mNSC and PEAKQC scores, revealed a lack of correlation between the two scores (Supplemental Fig. S4), implying that they assess different aspects of the data.

Analogous to the evaluation of PEAKQC, we conducted three iterative analyses utilizing the mNSC filtering approach. This time we assessed intervals (Fig. 2f) with defined minimum and maximum thresholds, due to the non-linear relationship between score and FLD quality. The first interval retained ~10 000 cells, organized into one large and several small clusters, the majority of which showed minimal overlap with CATLAS. The second interval also encompassed ~10 000 cells, yielding a UMAP embedding comparable to that produced using PEAKQC-based criteria, characterized by peripheral clusters encompassing a larger fraction of the reference population. The third filtering iteration captured a substantially greater number of cells (~50 000) and exhibited a pattern similar to that of the initial iteration, characterized by one predominant, several smaller clusters, and minimal overlap with the reference.

These results underscore the complexity of mNSC-based filtering, which necessitates manual intervention to identify an optimal threshold. In contrast, PEAKQC scores provide a more direct and consistent method for identifying high-quality cells.

### QC benchmarking and parameter evaluation

Subsequently, we extended our analysis to the wider perspective of scATAC-seq QC and performed a benchmark analysis across multiple reference subsets.

To mitigate potential bias arising from reliance on the CATLAS reference, which may reflect the authors’ specific processing strategy, we developed an additional metric to assess embedding quality following cell filtering. This metric assumes that high-quality data reflect biological features rather than technical variability (namely cell types), thereby shaping embeddings and clustering accordingly. High-quality cells are expected to cluster by feature similarity, whereas low-quality cells may yield biologically less meaningful, diffuse patterns of lower density. The proposed metric, termed the *distance score,* evaluates cell quality in a UMAP [35] embedding by assessing the consistency of their feature sets relative to their spatial positions. To achieve this, we compute the Levenshtein distance, quantifying the number of feature alterations required to transform one cell’s feature set into that of another. To accommodate varying feature counts across cell pairs, this distance is normalized by the total number of features. Cells are compared based on the Euclidean distance in the embedding space. For each cell, we define two cohorts: (1) nearest neighbors and (2) distant cells, based on Euclidean distances. We hypothesize that a high-quality embedding is characterized by low Levenshtein distances among neighbors and large ones among distant cells. Mean Levenshtein distances are calculated for each cohort. The score for a cell is derived by summing the mean distances for neighbors and the reciprocal of the mean distances for distant cells. Lower scores indicate higher quality. The dataset level score is defined as the median across all cells. This distance score is subsequently employed to rank the analysis runs performed below.

We benchmarked TSSe, FRiP, total counts (total number of unique fragments, TC), mNSC, and PEAKQC scores on sub-tissues from CATLAS, specifically IOBHN, left heart ventricle (Sample ID: IOBHO), and gastrocnemius medialis (Sample ID: ADA6L). Filtering was performed multiple times with varying thresholds using both individual and combined metrics, resulting in 144 distinct analysis runs. Evaluation criteria included (i) gold standard reference filtering coverage, (ii) percentage of additional non-reference cells, (iii) visual inspection of embeddings, (iv) total number of remaining cells, and (v) distance score. A comprehensive table of results is provided in Supplemental Table S2. The primary objective was to retain as many high-quality cells as possible by minimizing the distance score while maximizing both reference coverage and the number of retained cells. The following section presents exemplary results for the IOBHN tissue, all benchmarks are summarized at the end.

**Figure 3:**
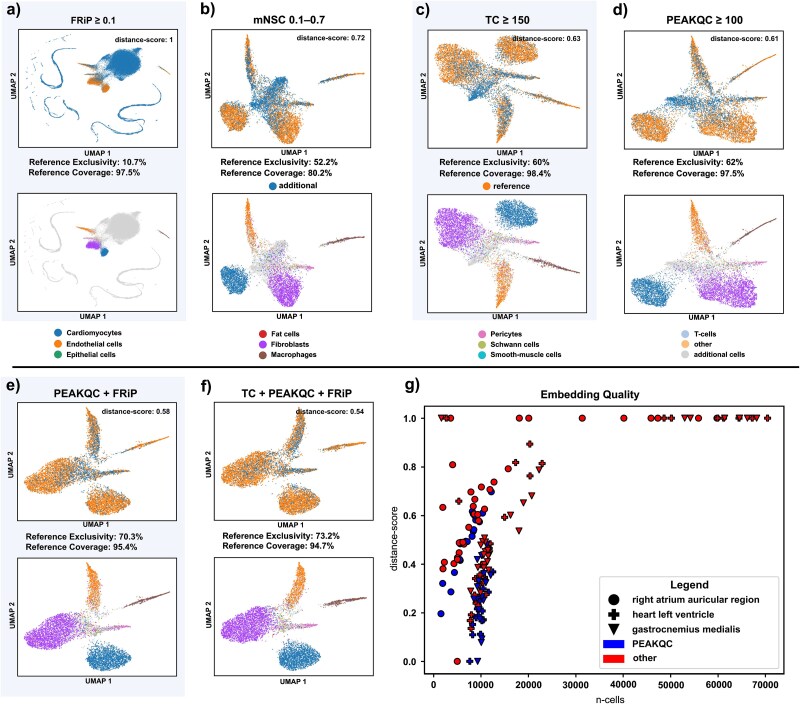
Embeddings and reference. (a–d) UMAP embeddings of data filtered by a single metric annotated with cells of the reference. Reference coverage and reference exclusivity are shown below the UMAP plots. The second row of UMAPs indicates the same cell filtering, but annotated with the cell type ontologies of the reference. (e and f) UMAP embeddings of data filtered by a combination of different metrics and annotated as in (a–d). (g) Scatterplot summarizing results of all performed runs across three tissues. The *Y*-axis represents distance score, while *X*-axis denotes number of cells retained after filtering. The shape of data points indicates tissues, while color denotes application of PEAKQC score for filtering.

Initial filtering was conducted utilizing individual metrics (Fig. 3a–d). Based on the distance score and cell counts, PEAKQC attained the highest ranking, followed by TC. Both methods also produced superior embeddings, characterized by well-defined cluster separation, which could be largely annotated to cell types in the reference (Fig. 3c and d).

Then we employed a filtering strategy that incorporated combinations of two metrics. This approach yielded improved distance scores, albeit at the expense of a reduction in the total number of retained cells. Interestingly, visual inspection of the embedding revealed that combinations involving signal-to-noise ratio metrics (e.g. FRiP, TSSe) reduced cells located between well-defined clusters, consequently increasing reference coverage, as these inter-cluster cells were mostly absent from the reference. Notably, our results indicated that the effects of FRiP were more pronounced at lower threshold values, whereas TSSe required stricter thresholds to achieve comparable outcomes. Consequently, we observed improved results in the embedding, leading us to conclude that the optimal combination of metrics was TC (≥150) and FRiP (0.1–2), which retained 9479 cells. This was closely followed by a combination of PEAKQC score and FRiP (Fig. 3e).

Finally, three-metric combinations yielded refined outcomes compared to two-metric combinations, albeit with a notable decrease in cell retention. Notably, the combination of PEAKQC score (≥100), FRiP (≥0.1), and TC (≥150) yielded the most favorable results, retaining 8475 cells and achieving a reference coverage of 94.7%. This particular combination resulted in the formation of distinct clusters with minimal inter-cluster connectivity (Fig. 3f).

Across all 144 filtering combinations on both datasets, we observed significant variability driven by different metrics and threshold settings. Notably, each individual metric removed distinct subsets of cells, underscoring the additive benefits of combining multiple filtering approaches. Our findings indicate that filter combinations incorporating the PEAKQC score yielded the most favorable results. This conclusion is substantiated by (i) an examination of the cell numbers retained and (ii) the distance scores achieved across all benchmark runs (Fig. 3g). While more stringent filtering decreased the total number of cells, quality measured by the distance score improved linearly as soon as PEAKQC was included.

To test transferability, we applied PEAKQC to two 10× Genomics datasets [33]: the atac_pbmc_5k_nextgem dataset, obtained from human peripheral blood mononuclear cells, and the 8k_mouse_cortex_ATACv2_nextgem_Chromium_X dataset, derived from mouse cortex tissue. Data were processed as described for CATLAS. We employed automated cell type annotation to evaluate the recovery of biologically relevant clusters following QC filtering. Notably, we recovered distinct, well-defined cell type clusters in both datasets (Supplemental Fig. S5). These results demonstrate the versatility of our filtering metric in accommodating diverse library preparation strategies.

### Rare cell types

**Figure 4:**
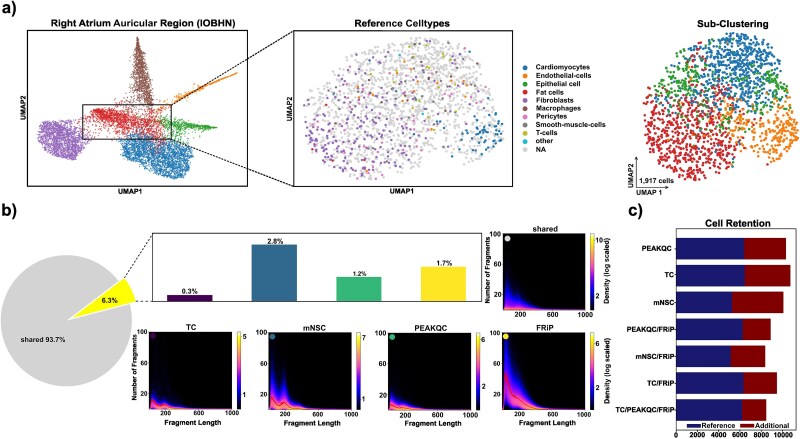
Embedding quality scatterplot. (a) UMAP embedding of IOBHN tissue (left); UMAP embedding of a subset of the original data (middle) annotated by cell types present in the reference and reclustered (right). (b) Pie chart displaying the distribution of filtered cells, with slices representing proportion of cells filtered by multiple metrics (93.7%) and those filtered exclusively by a single metric (6.3%). Density plots of FLDs for single-cell subsets corresponding to cells filtered by a single metric, displayed alongside a bar plot showing the fractions. (c) Barplot of cells after filtering that were also retained in the reference or removed in the reference.

While it was possible to recover the more abundant cell types from the reference as distinct clusters in our embeddings, this was not the case for rare cell types. Instead, these cell types were mostly scattered among other cells absent in the reference and accumulated in a central cluster within our embeddings (Fig. 3b–d). To explore whether rare reference cell types or additional cells introduced by the three metrics would improve clustering or annotation, cells were subsetted from the well-annotated clusters and a new embedding and clustering was generated (Fig. 4a, PEAKQC embedding).

It is important to note that the PEAKQC metric was the only one to reveal distinct cell type substructures (see Supplemental Fig. S6a). Specifically, a high density cluster was observed for fibroblasts, cardiomyocytes, endothelial cells, and T-cells, among other cell types. In contrast, the cell type distribution appeared largely random when using other metrics (see Supplemental Fig. S7). To further test the new cells for marker positions, we annotated all peaks using the Panglao cell type marker database [36]. As shown in Supplemental Fig. S6b, we found enriched marker accessibility for the respective cell types. Notably, the total number of peaks overlapping with Panglao markers in the entire dataset was limited.

In conclusion, our results demonstrate that cell filtering based solely on PEAKQC reintroduces cells that were excluded by the conventional gold standard filtering approach. Moreover, the inclusion of these additional cells enhances the identification and grouping of highly abundant and rare cell types.

### QC-metric specificity

Finally, we aimed to quantify and qualify differences between the investigated metrics and examine their effects on the overall FLD. We isolated cell sets that were either filtered exclusively by one, or more than one of the metrics. The thresholds applied in this analysis corresponded to the optimal values identified previously (Fig. 3f). As shown in Fig. 4b, the largest proportion of cells discarded from all available cell events was shared among multiple metrics, indicating that these cells satisfied filtering criteria for at least two metrics. This group was substantial and accounted for ~94% of all cell events. For these ‘shared’ detected cells, we observed very low mean fragment counts and a poorly defined periodic pattern (Fig. 4b, right). This finding is expected, as the large majority of these cells own very few fragments, have high PCR amplification rates, and other technically driven noises, which were clearly detectable by multiple metrics.

Among metric-specific subsets of cells (accounted for ~6%), mNSC removed the largest exclusive fraction (1784 cells). Interestingly, these cells exhibited a strong periodic pattern and relatively high fragment counts compared to the shared cells. The second largest fraction of cells was exclusively removed by FRiP. These cells displayed the highest fragment counts among subsets, but only showed a faint nucleosomal pattern. Cells exclusively removed by PEAKQC score formed the third largest fraction and showed relatively low fragment counts with a weak periodic pattern. TC removed the fewest unique cells, which nevertheless exhibited clear periodicity.

Next, we quantified the overlap with the reference among cells retained by each metric (Fig. 4c). We observed that TC, PEAKQC, and their combinations, including those involving FRiP, resulted in the highest overlap with the reference dataset. These results align with earlier findings, demonstrating >90% reference coverage for these filters. In contrast, mNSC and its combinations captured fewer cells from the reference. Notably, mNSC also accounted for the largest number of additional cells, followed by TC and PEAKQC, whereas combinations involving FRiP resulted in lower numbers of additional cells.

In conclusion, we found the proportion of cell events exclusively removed by shared metrics, FRiP, or PEAKQC to generally exhibit poorly defined periodic patterns suggesting correct identification of low-quality cells, while retaining cell type diversity.

## Discussion

While the FLD is a commonly employed metric for evaluating the quality of bulk ATAC-seq libraries, its use in the context of scATAC-seq has been under investigated [10, 12]. PEAKQC offers seamless integration of a FLD-based quality metric, enabling a more comprehensive evaluation of library quality. Our results demonstrate that PEAKQC can effectively filter cell events, yielding stable and reliable outcomes, and potentially supplanting existing quality metrics. The linear and robust nature of the PEAKQC score represents a noteworthy advantage, as it circumvents the need for the definition of upper and lower cutoffs. PEAKQC involves the convolution of a single wavelet with FLD tracks. This approach enhances the detection of genuine signals that may otherwise be obscured by noise. Unlike other methods, PEAKQC assumes that the desired frequency is inherently present within the available fragments eliminating high scores originating from other factors.

Nonetheless, it is important to emphasize that both technical and biological factors significantly influence the manifestation of FLD characteristics. From a technical standpoint, the Tn5 to nucleus ratio and incubation time are particularly relevant, as they have been observed to substantially impact the FLD pattern [5]. When either of these is extended, a disproportionate number of short DNA fragments are produced, while the number of fragments spanning nucleosomes is significantly reduced [5, 12]. Conversely, low ratios of Tn5 lead to a decreased signal-to-noise ratio [15]. Additionally it was shown that high cluster density within a sequencer lane favors shorter fragments [15]. Furthermore, it has been speculated that an improper ratio of magnetic beads to DNA concentration can lead to a biased size selection during library preparation and thus to alterations in the FLD [12]. From a biological perspective, our findings suggest that the underlying cell type or tissue may contribute to the overall strength of the FLD pattern (Supplementary Fig. S1). We hypothesize that differentiated cell types exhibit a more stable periodical pattern compared to less differentiated cells, although further analysis is required to validate this observation. Within this study, especially when analyzing CATLAS cohorts IOBHN and IOBHO, we were not able to pinpoint differences in mean FLD scores linked to specific cell types (Supplemental Fig. S2). It is important to acknowledge that the FLD score depends on the number of fragments available. Although substantial disparities at the higher end of the fragment count distribution are mitigated through our internal downsampling procedure, cells possessing fewer fragments than the specified downsampling threshold may be assigned artificially to low scores. However, this effect is likely to be minimal, since cells with a low fragment count are generally removed during QC, even without FLD assessment.

While the PEAKQC score positively correlated with a periodical pattern in the FLD, mNSC appeared to confound sequencing depth and TC with cell quality. This is attributed to the dependency of mNSC on the fraction of different read lengths, rendering it highly unstable in cases of low fragment counts. This underscores the importance of using mNSC in conjunction with filtering based on fragment or feature counts. In contrast, PEAKQC incorporates a correction for sequencing depth.

QC for single-cell ATAC-seq is inherently complex, since optimal thresholds vary across samples and different QC metrics exert distinct filtering effects. Our analysis indicates that PEAKQC, alone or in combination with other metrics, yields stable results across varying thresholds. Notably, combining PEAKQC or TC with FRiP achieves superior outcomes, since this pairing effectively removes low-quality cells. Interestingly, we observed that cells removed exclusively by FRiP exhibited only faint nucleosomal patterns, suggesting that FRiP adds unique value to the filtering process by addressing additional quality aspects.

## Conclusion

PEAKQC extends the utility of FLD as a metric to identify high-quality single cells. By providing insights into experimental settings and errors, PEAKQC complements standard QC workflows. Extensive benchmarking highlights its ability to unify multiple aspects of QC, leading to robust and reliable results.

## Materials and methods

### Preprocessing and basic analysis of scATAC-seq data

CATLAS raw FASTQ files were preprocessed by alignment to hg38 [37] using the Burrows–Wheeler Aligner [38]. The resulting BAM [39] file was sorted, indexed, and filtered to remove PCR duplicates using pysam [40]. A fragments BED file was generated with sinto [41]. Peaks were called via MACS2 [42]and used to generate a binned matrix with a bin size of 5000 bp using SnapATAC [18]. The Snap object was converted to an AnnData object. For experimental and benchmarking procedures we utilized the sctoolbox [43]. This framework facilitates comprehensive single-cell data analysis for both scATAC-seq and single-cell RNA-seq data. In this study, we used sctoolbox for key analysis steps, including AnnData assembly, QC scoring and filtering, normalization, dimensionality reduction, component subsetting, clustering, and cell type annotation. An associated set of notebooks is available via the PEAKQC repository [44].

### Implementation of scATAC-seq QC metrics

#### TSSe calculation

The TSSe score was implemented in sctoolbox [43] as described by ENCODE [45]. Its calculation involves aggregating fragments around all TSSs for each single cell, extending ±2000 bp from each TSS. To account for potential biases, the mean fragment coverage within the outer 100 bp flanking regions is computed and used as a correction factor. The final TSSe score for each cell is then determined by calculating mean fragment coverage within a ± 50 bp window centered on the TSS.

#### FRiP score calculation

The FRiP score is implemented within the single-cell framework and is calculated by determining the fraction of reads that fall within called peaks. Previous studies have demonstrated that the FRiP score is positively correlated with the number of detected features [46].

#### Nucleosome signal of muon

The mNSC implemented by Muon is based on the assumption that nucleosome-free DNA results in ATAC-seq fragments shorter than 147 bp, while mononucleosomal DNA creates fragments of 147-294 bp. The mNSC score is calculated as the ratio between these two fragment length categories [27].

#### PEAKQC implementation

PEAKQC implements a discrete frequency analysis based on convolution and is inspired by wavelet transform techniques. This approach enables the detection of underlying periodicities in the FLD while avoiding the limitations of conventional peak calling, which is insensitive to overlapping patterns. Furthermore, to minimize the impact of sequencing depth on the analysis, PEAKQC subsamples the fragments per cell to a uniform number, prior to convolving the FLDs with a single wavelet. The wavelet function consists of a centered sine wave with a wavelength of 150 bp, modulated by a Gaussian envelope Equation (1):


(1) \begin{equation*} {\psi}_{a,b}(t)=\cos \left(a\left(t-b\right)\right)\ast \left(\frac{1}{\sigma^{\ast}\sqrt{2\pi }}\right)\exp \left(-\frac{1}{2}\frac{{\left(a\left(t-b\right)\right)}^2}{\sigma^2}\right) \end{equation*}

To optimize performance, we conducted parameter tuning for both the wavelet wavelength and the Gaussian smoothing parameter, sigma. This involved performing a full wavelet transform on multiple cells representing a range of FLD qualities. The transformation was repeated across a range of sigma values to assess its effect on signal clarity and noise suppression. We identified a wavelength of ~150 bp and a sigma value of 0.4 as the most effective combination for denoising periodic signals in the FLDs while minimizing false positives. Notably, this parameter set satisfies the wavelet admissibility condition. Deviations from these values impaired performance: wavelengths substantially above or below 150 bp failed to capture the periodic signal, while variations in sigma affected sensitivity: lower values weakened signal strength, whereas higher values increased the risk of false positives. Following convolution with the wavelet, peak calling is performed on the transformed signal to determine relevant nucleosome positions. Identified peaks Equation (2) are then weighted using a scoring mask Equation (3), which encodes the expected nucleosomal pattern as a probability distribution derived from normal distributions. Probabilities were generated by peak calling on a series of high-quality bulk FLD patterns, which confirmed the nucleosomal pattern described in the literature. Of note, in cases where the nucleosomal pattern strongly deviates from the ‘normal’ pattern (e.g. due to a very different library architecture), we suggest adapting the PEAKQC scoring mask:


(2) \begin{equation*} \mathrm{Peak}\kern0.17em \mathrm{amplitudes}\;P\left(x,{x}_i^{\hbox{'}}\right)=\sum \limits_{i=0}^4{a_i}^{\ast}\delta \left(x-{x}_i^{\hbox{'}}\right) \end{equation*}

(3) \begin{equation*} \mathrm{Score}\kern0.17em \mathrm{mask}\;S\left(x,{x}_i\right)=\sum \limits_{i=0}^4\frac{1}{\sigma \sqrt{2\pi }}{e}^{-\frac{1}{2}{\left(\frac{x-{x}_i}{\sigma}\right)}^2} \end{equation*}

Finally, weighted peaks are summed to compute the final quality score Equation (4):


(4) \begin{equation*} \mathrm{FLD}\;\mathrm{score}=\int \sum P\left(x,{x}_i^{\hbox{'}}\right)\ast S\left(x,{x}_i\right) dx=\sum{a_i}^{\ast }S\left({x}_i^{\hbox{'}},{x}_i\right) \end{equation*}

This process is repeated for each individual cell, enabling scoring on a single-cell scale.

**Key Points**

- Quality control of scATAC data identifies high-quality cells prior to data interpretation.
- There is no firmly defined best practice for quality control, and a standard measure for scATAC cell quality is not established.
- PEAKQC calculates a new metric for cell quality based on the library fragmentation pattern, which improves on current visual approaches.
- The PEAKQC score scales linearly and robustly, simplifying cutoff definition (higher = better).
- Extensive comparison to other metrics and metric combinations in multiple datasets demonstrate performance of PEAKQC and suggest it as a new standard to the field

**Conflict of interest**: We declare that we have no conflicts of interest related to this publication. There are no financial, personal, or professional relationships that could influence or bias the content presented.

## Supplementary Material

Supplemental_1_bbaf465

Supplemental_2_new_order_bbaf465

Supplemental_3_bbaf465

Supplemental_4_new_order_bbaf465

Supplemental_5_new_order_bbaf465

Supplemental_6_new_order_bbaf465

Supplemental_7_new_order_bbaf465

Supplemental_table_1_bbaf465

Supplemental_table_2_bbaf465

## Data Availability

The CATLAS dataset used in this article is available by the Gene Expression Omnibus (GEO) and can accessed with GSE184462. The 10x Genomics datasets used in this article is available by 10x Genomics at https://www.10xgenomics.com/datasets, and can be accessed with the IDs **a)** atac_pbmc_5k_nextgem (PBMCs from from a healthy donor (Human), single cell ATAC-seq dataset by 10x Genomics, (2021, Mai 03). https://www.10xgenomics.com/datasets/5-k-peripheral-blood-mononuclear-cells-pbm-cs-from-a-healthy-donor-next-gem-v-1-1-1-1-standard-2-0-0 (accessed 2025, September 5)) **and b)** 8k_mouse_cortex_ATACv2_nextgem_Chromium_X (8K Mouse Cortex Cells from BrainBits, single cell ATAC-seq dataset by 10x Genomics, (2022, March 29) https://www.10xgenomics.com/datasets/8k-adult-mouse-cortex-cells-atac-v2-chromium-x-2-standard (accessed 2025, September 5)). Fig. 1a–d Created in BioRender. Detleffsen, J. (2025) https://BioRender.com/ y85q94.
