# Supplementary figures and images for: PEAKQC: periodicity evaluation in single-cell ATAC-seq data for quality assessment
Source: Brief Bioinform. 2025 Sep 16;26(5):bbaf465. doi: 10.1093/bib/bbaf465 (PMC12448717; doi:10.1093/bib/bbaf465)

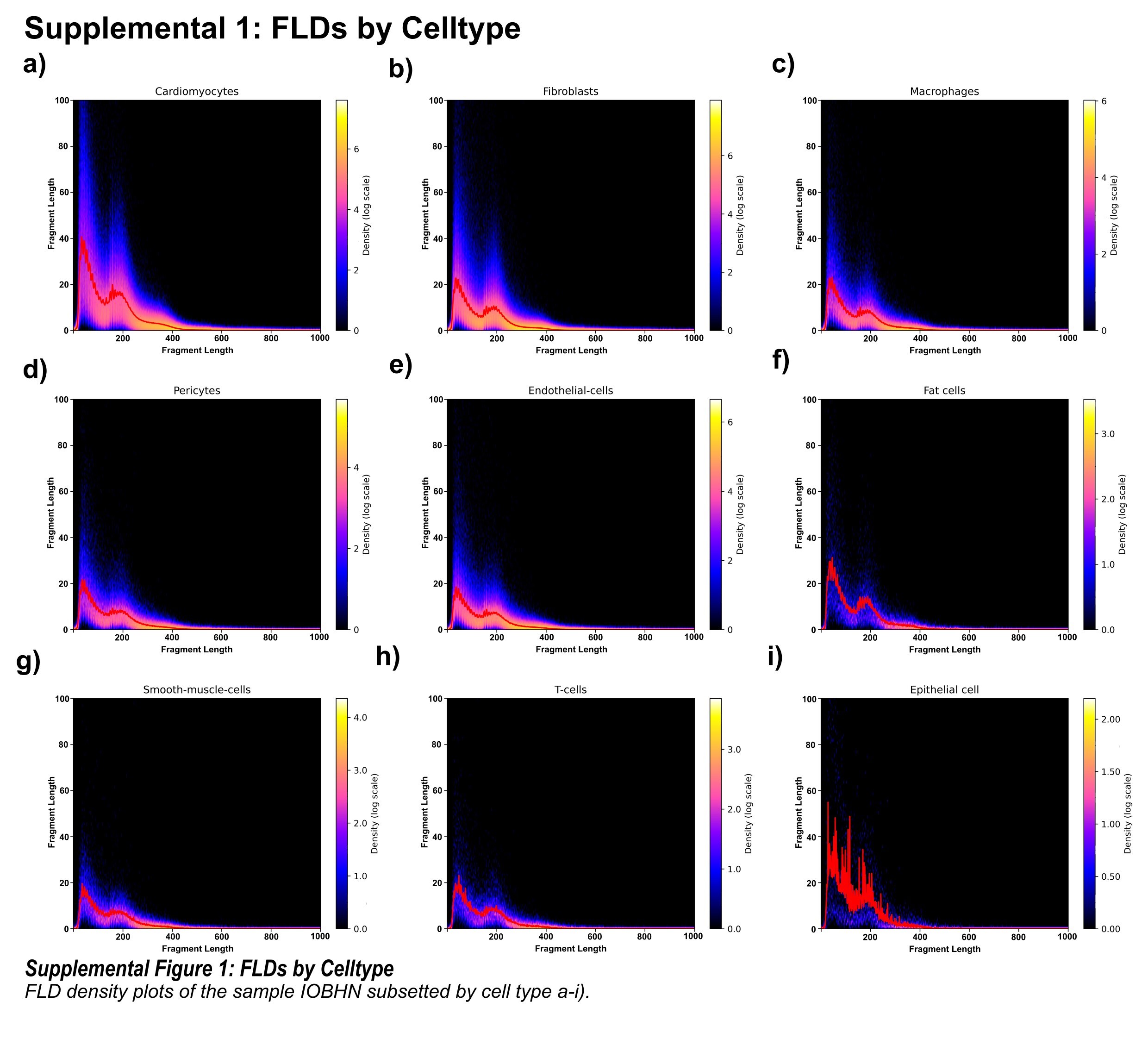

Supplement: Supplemental_1_bbaf465 [file supplemental_1_bbaf465.jpeg]

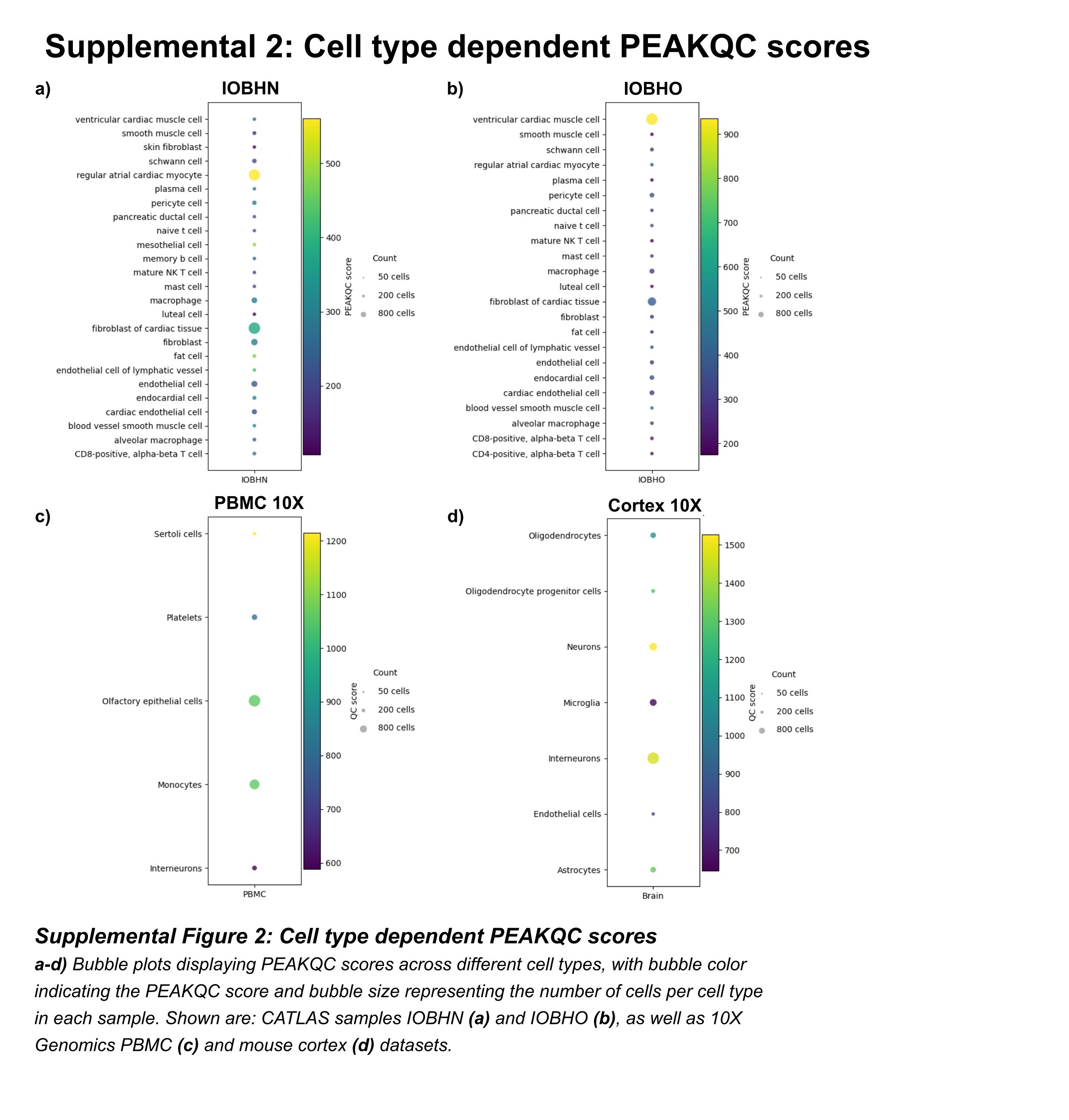

Supplement: Supplemental_2_new_order_bbaf465 [file supplemental_2_new_order_bbaf465.jpeg]

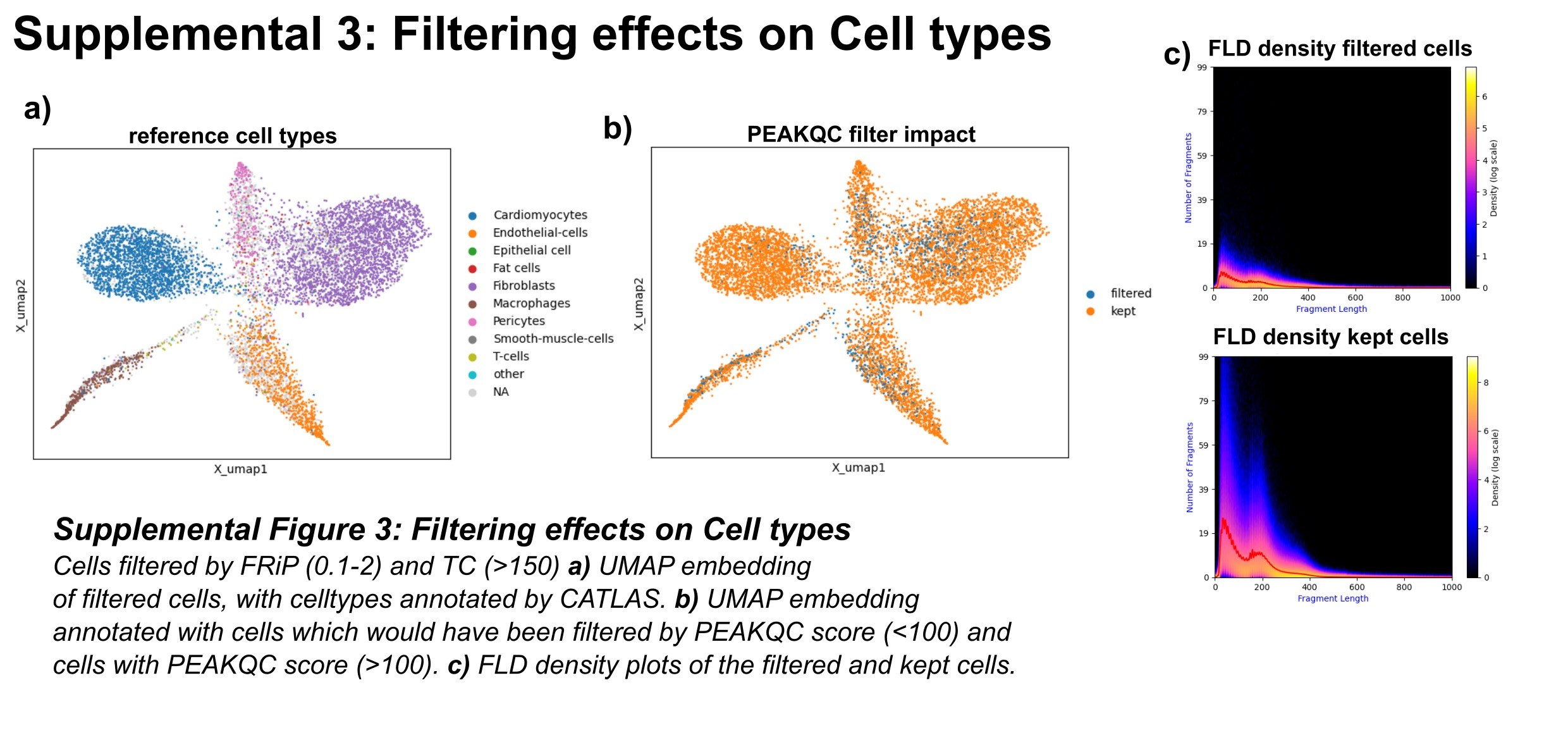

Supplement: Supplemental_3_bbaf465 [file supplemental_3_bbaf465.jpeg]

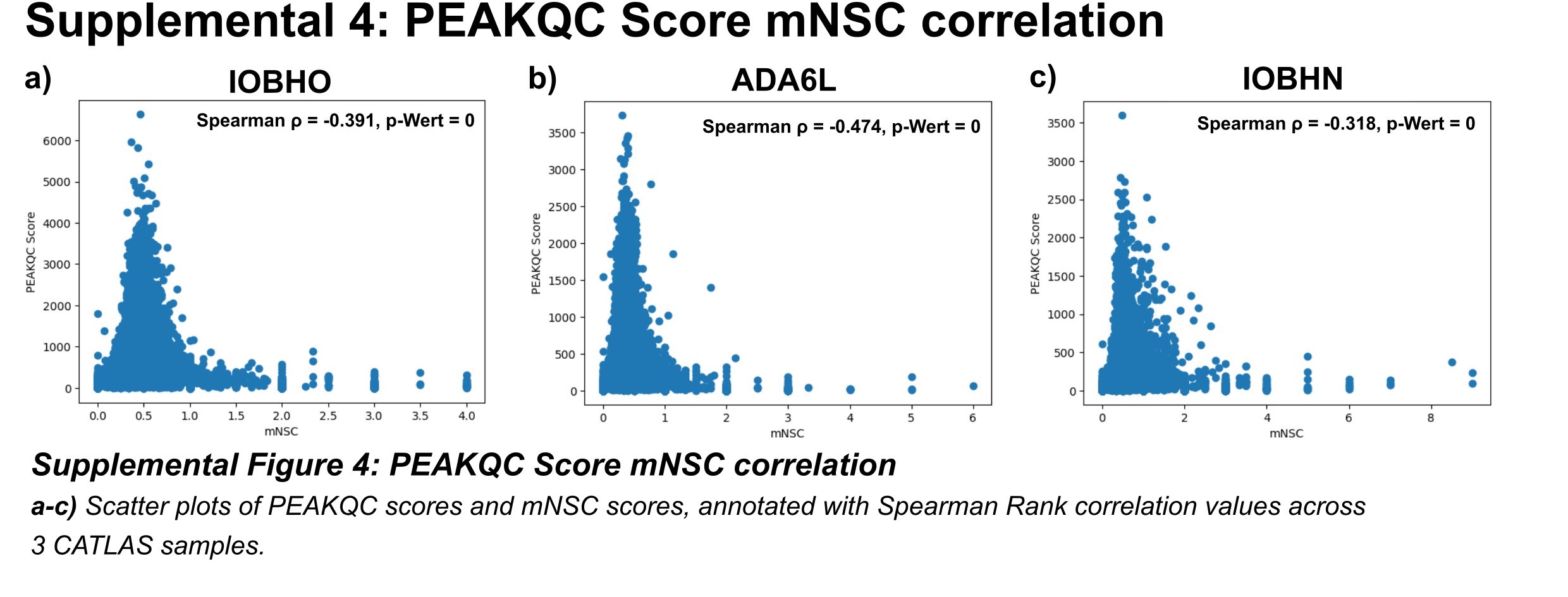

Supplement: Supplemental_4_new_order_bbaf465 [file supplemental_4_new_order_bbaf465.jpeg]

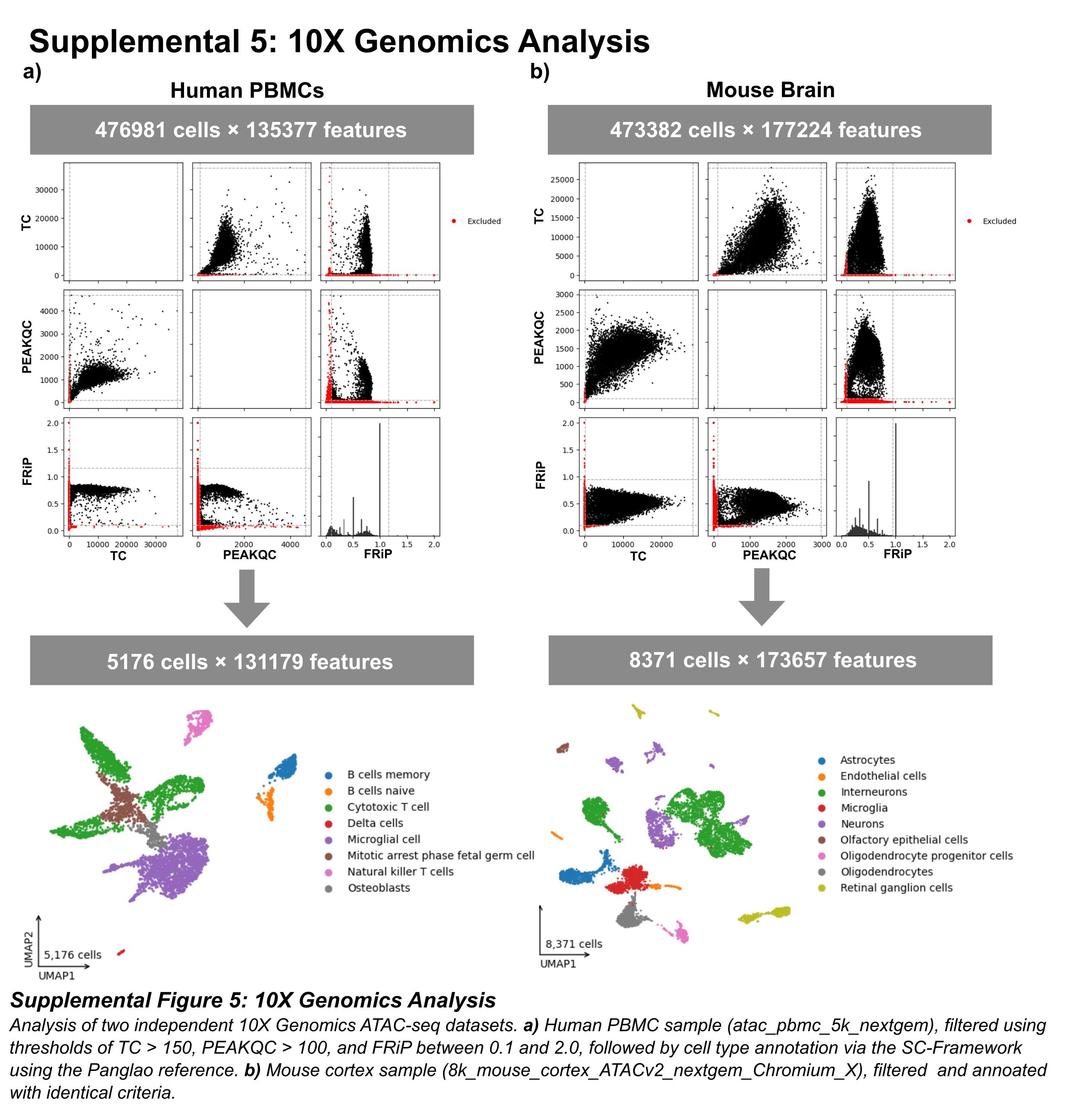

Supplement: Supplemental_5_new_order_bbaf465 [file supplemental_5_new_order_bbaf465.jpeg]

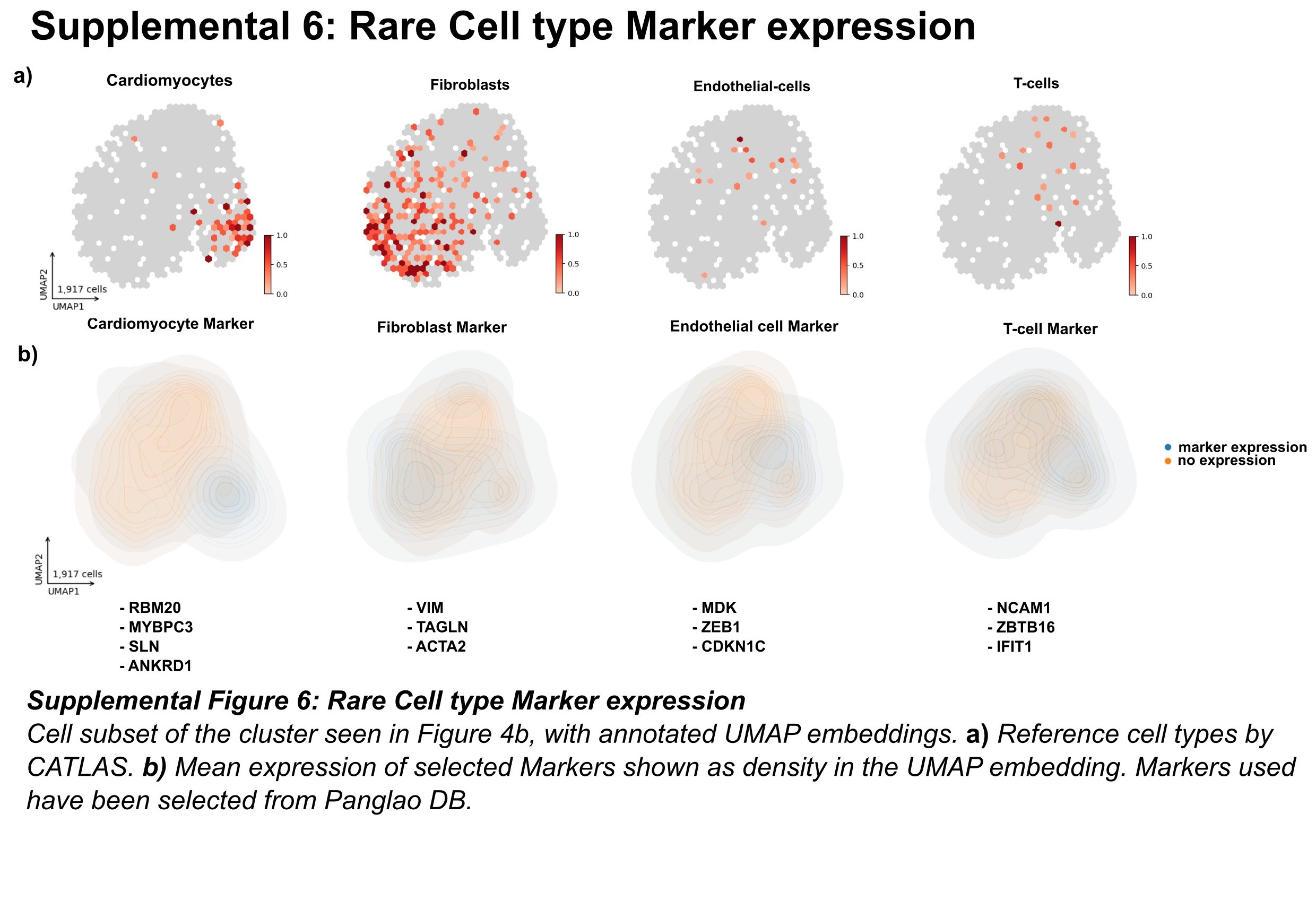

Supplement: Supplemental_6_new_order_bbaf465 [file supplemental_6_new_order_bbaf465.jpeg]

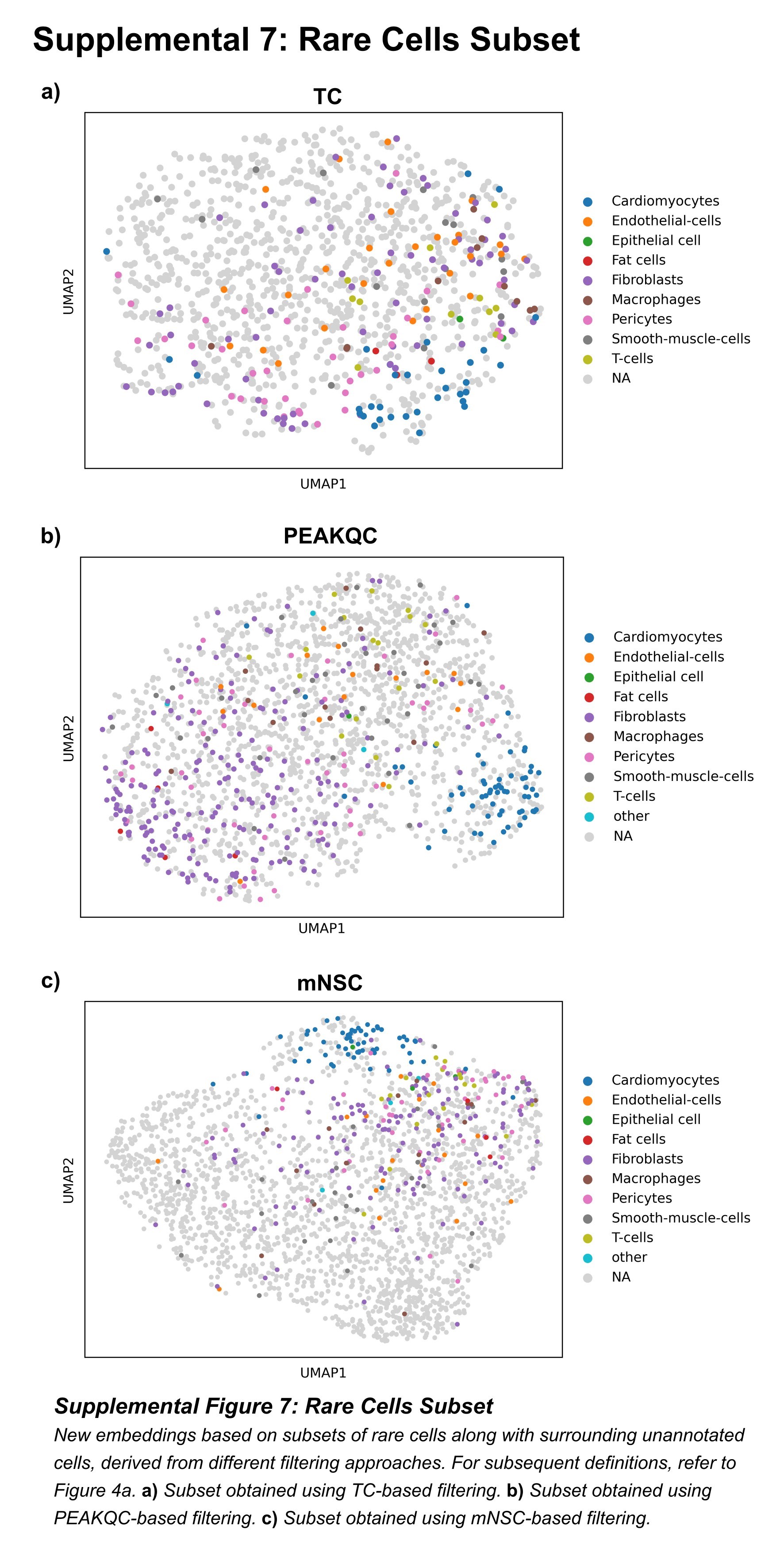

Supplement: Supplemental_7_new_order_bbaf465 [file supplemental_7_new_order_bbaf465.jpeg]
